# ‘Simulation-based learning in psychiatry for undergraduates at the University of Zimbabwe medical school’

**DOI:** 10.1186/s12909-015-0291-8

**Published:** 2015-02-21

**Authors:** Angharad Piette, Florence Muchirahondo, Walter Mangezi, Amy Iversen, Frances Cowan, Michelle Dube, Hugh Grant- Peterkin, Ricardo Araya, Melanie Abas

**Affiliations:** 1 Institute of Psychiatry, Psychology and Neuroscience, King’s College London, London, UK; 2 Department of Psychiatry, University of Zimbabwe College of Health Sciences, Harare, Zimbabwe; 3 University College London, London, UK; 4 London School of Hygiene and Tropical Medicine, London, UK

**Keywords:** Simulation, Medical education, Depression, Psychiatry, Mental health, Low-income countries, Developing countries, Africa

## Abstract

**Background:**

The use of simulated patients to teach in psychiatry has not been reported from low-income countries. This is the first study using simulation teaching in psychiatry in Africa. The aim of this study was to introduce a novel method of psychiatric teaching to medical students at the University of Zimbabwe and assess its feasibility and preliminary effectiveness. We selected depression to simulate because students in Zimbabwe are most likely to see cases of psychoses during their ward-based clinical exposure.

**Methods:**

Zimbabwean psychiatrists adapted scenarios on depression and suicide based on ones used in London. Zimbabwean post-graduate trainee psychiatrists were invited to carry out the teaching and psychiatric nursing staff were recruited and trained in one hour to play the simulated patients (SPs). All students undertaking their psychiatry placement (n = 30) were allocated into groups for a short didactic lecture on assessing for clinical depression and then rotated around 3 scenarios in groups of 4–5 and asked to interview a simulated patient with signs of depression. Students received feedback from peers, SPs and facilitators. Students completed the Confidence in Assessing and Managing Depression (CAM-D) questionnaire before and after the simulation session and provided written free-text feedback.

**Results:**

Post-graduate trainers, together with one consultant, facilitated the simulated teaching after three hours training. Student confidence scores increased from mean 15.90 to 20.05 (95% CI = 2.58- 5.71) t (20) = 5.52, (p > 0.0001) following the simulation teaching session. Free-text feedback was positive overall with students commenting that it was “helpful”, “enjoyable” and “boosted confidence”.

**Conclusions:**

In Zimbabwe, simulation teaching was acceptable and could be adapted with minimal effort by local psychiatrists and implemented by post-graduate trainees and one consultant, Students found it helpful and enjoyable and their confidence increased after the teaching. It offers students a broader exposure to psychiatric conditions than they receive during clinical attachment to the inpatient wards. Involving psychiatry trainees and nursing staff may be a sustainable approach in a setting with small number of consultants and limited funds to pay for professional actors.

## Background

Simulation is used across the world in medical education to expose students to real-life clinical scenarios without exposing patients to risk or discomfort [1]. The use of simulated patients, whereby a ‘plant’ such as a health professional or actor, is coached to present certain signs and symptoms, has increased in psychiatry education worldwide [2] and offers a way of ‘moving the learning curve away from the patient’ [3]. Simulation has been shown to be more enjoyable and effective in improving clinical skills than didactic teaching [1,4]. Simulation can be used to overcome variable experience and lack of exposure to certain conditions encountered with clinical attachments [5] and provides the opportunity to present rare, complex or anxiety-provoking scenarios to students in a safe and contained environment.

There is a dearth of literature on the use of simulated patients in medical education in Africa. One possible reason for this is that the development of simulated teaching scenarios including recruitment and training of simulated patients is considered time and resource-intensive [6,7]. Another is the impact of migration of senior doctors away from sub-Saharan Africa, resulting in low numbers of academic medical educators [8].

Zimbabwe is recovering from a period of extreme economic instability [9]. This has resulted in limited resources across the public services and mass emigration of professionals to high income countries [8]. This has impacted upon Zimbabwe’s medical school as a whole, with the Department of Psychiatry being particularly affected by depleted faculty. Under these conditions, development of medical education and the psychiatry curriculum for undergraduates has been in stasis for the past two decades. Teaching is primarily didactic and is based on the curriculum of a British medical school, developed in the 1980s.

Zimbabwe has five consultant psychiatrists working in the public sector, including 3 consultants in the Department of Psychiatry at the University of Zimbabwe. The public mental health service serves the country’s population of 13 million people although most of the specialist psychiatric services are based in Zimbabwe’s capital city Harare. There are 2 psychiatric hospitals in Harare, with around 70 psychiatric inpatients on the acute wards, where the average length of stay is around 2 weeks. There are also 4 psychiatric outpatient clinics per week with around 80 patients being seen in each clinic. The consultant psychiatrists are responsible for these clinical services as well as teaching undergraduates and postgraduates in psychiatry. There are around 150 medical students in each year at the University of Zimbabwe who all undertake a six-week psychiatry placement in their 4th year.

Depression is the commonest psychiatric disorder in Zimbabwe [10,11] with the highest disease burden of the mental disorders worldwide [12]. This is compounded by the effects of HIV (prevalence rate 14.90% [13]), both in terms of psychological and social impacts and complex neuropsychiatric complications. However, student exposure to common mental disorders such as depression and anxiety during their psychiatry placement is limited as most of these patients present in primary care or private practice. In a survey in 2010, only 29% of medical students rated their education in the common mental disorders as good or very good.

Undergraduates spend most of their clinical attachments on the acute inpatient wards or psychiatric outpatient clinics where the majority of patients are suffering from psychotic illnesses, often with physical comorbidities and complex risk issues. Most students will receive no further specialist training in psychiatry following this placement. It is important, therefore, that they have a sound grasp of diagnosis and basic management of the common mental disorders, particularly anticipating their time spent in rural hospitals where they are posted immediately after graduating and are responsible for delivering all mental health services.

### The setting

The Medical Education Partnership Initiative (MEPI) program was launched in 2010 with the aim of improving medical education and increasing retention of health workers in Africa [14,15]. In MEPI, African medical schools lead programs of medical education, supported by local and international partners. The aims are to increase numbers of healthcare professionals, improve retention of trained professionals, build capacity of existing faculty [14,16] and strengthen locally relevant research [17].

Improving Mental Health Education and Research Capacity in Zimbabwe (IMHERZ) is the only program specific to mental health in the MEPI network that was funded in the first round of the program. As part of IMHERZ activities, the undergraduate psychiatry curriculum at the University of Zimbabwe was developed in collaboration with external partners.

Students at the University of Zimbabwe College of Health Sciences receive the majority of their psychiatry tuition during their 6-week placement in the 4th year. This consists of a week of didactic lectures followed by five weeks of clinical attachment to the psychiatric wards and weekly case presentations seminars within the department.

In this study, we describe the introduction of a novel teaching intervention to improve student exposure to depression, which was designed to be feasible and sustainable in a low- income setting. Specifically, we aimed to discover if a 2-hour simulation session improves student confidence in assessing and managing depression and to assess the effect on student enjoyment.

## Methods

We present a mixed methods study. Our primary outcome of interest was a change in student confidence measured between 2 time points, before and after a simulation-based teaching session. The secondary outcome was student acceptability and enjoyment, which was assessed by analysis of free-text responses to a written survey administered after the teaching session.

### Participants

All 4th year students (n = 30) on their psychiatry block at the University of Zimbabwe in January to March 2013 were included. All the students were Zimbabwean in nationality and were aged between 20 and 26 years (mean 22.9, SD 1.7). There were 7 female students and 23 male. All 30 students provided written consent for their questionnaire responses to be used in the study.

23 students took part in the simulation sessions. The 7 students who missed the session were on attachment at a different hospital site and were not able to attend due to transport problems on the day.

### Developing the scenarios

The design of the sessions was adapted from the ‘Extreme Psychiatry’ approach developed in the UK where professional actors play simulated patients with student teaching facilitated by a team of consultants and senior trainees [18]. Pre-pilot work took place, in Zimbabwe, whereby ten volunteer students took part in role-play sessions of three scenarios on depression with feedback from their peers and from the facilitator. They also provided feedback on the scenarios and completed confidence questionnaires before and after the session. All students reported an increase in confidence in assessing and managing depression after the session and free-text feedback was positive overall. Local psychiatrists modified the scenarios following the pre-pilot session, altering details and demographic data to make the scenarios more realistic and culturally appropriate. For example, this led to the addition of an HIV and depression station. In addition, we added a scenario of a patient with prominent somatization symptoms, which is a common presentation of depression in Zimbabwe.

### A sample scenario used in the simulation teaching

Instructions for medical student

Esther Nondo is a 21 year old woman who lives with her mother in Harare. She was initially brought to the medical out patients with her mother for headaches. A history was taken and it was found that the headaches sounded like tension headaches. There were no other worrying medical features like dizziness, visual disturbances or seizures. She mentioned sleep disturbance. She also had issues with her boss’s daughter.

She was initially treated for depression with problem solving therapy. She explored the pros and cons of different approaches to dealing with the boss’s daughter. She decided to tell her mother who then visited the shop with her to talk to her boss. She also tried some behavioural activation (visited friends, started dancing), which helped and her mood improved.

She has now been brought to A and E nine months later, after breaking up with her boyfriend, she took 15 malaria tablets, it’s not clear if she wanted to die. She’s medically fit to go home.

You are the psychiatric junior doctor. You have been asked to see her before she’s sent home to see if she can be discharged.

Please take a history and assess for features of depression. The medical consultant will ask you some questions regarding management when you finish your assessment.

### Simulated patients

Limited resources available in our setting meant that employing professional actors was not possible. Instead, psychiatric nursing staff from the inpatient wards were recruited as SPs. Several training sessions took place in order to reach a number of different staff. The training consisted of running the scenarios with nurses playing the SP and members of faculty playing the student role. Feedback was then given to SPs by faculty members in attendance about how to keep their performance consistent and realistic, e.g. giving short and monosyllabic answers initially, becoming more forthcoming depending on the students’ communication style and questioning. These instructions were also printed on handouts as well as guidance on body language, eye contact etc. for the SPs.

### Intervention

Each session was run with 3 facilitators, who were a combination of senior faculty and postgraduate psychiatry trainees. Postgraduate trainees underwent a three-hour training session in facilitating the simulation sessions, which included guidance on facilitating peer feedback and group discussion as well as providing direct feedback to participants.

Two sessions took place on different days, with 14 students attending on the first day and 9 on the second.

The session lasted for two hours in total and began with a 20-minute didactic lecture. The lecture served as a refresher on the presentation of clinical depression and sensitive assessment of the depressed patient.

Students were then divided into groups of 4–5 and rotated around 3 different simulated patients (SPs) all playing different patients with features of depression. Attached to each SP was a facilitator, who was responsible for timing the scenario and providing feedback. The scenarios followed a similar structure but with different clinical themes;

1. A male patient with HIV and mood symptoms
2. A young woman presenting following an overdose in the Emergency Department
3. A woman with symptoms of depression related to infertility.

Within the small groups, 1 student volunteered to interview the SP and 3 of their peers were assigned roles in giving specific feedback; 1) was asked to take notes and comment on positive aspects of the interview, 2) another was asked to comment on aspects for improvement and 3) the third was asked to look out for one particularly good ‘golden moment’ or phrase used during the interview [18]. The simulated patient in each scenario also provided feedback, focusing on communication skills. Each scenario was ten minutes in length. After 9 minutes, the facilitator asked the student, “What is your differential diagnosis?” and asked to justify this, and “would you send this patient home?”. The answers to these questions were then discussed in the group during the feedback session.

The larger group came back together after rotating around all 3 scenarios to discuss the experience and review learning objectives. The session lasted for two hours in total.

## Results

### Measures

A questionnaire on Confidence in Assessing and Managing Depression (CAM-D) was developed based on literature on measuring self-efficacy [19] and work done previously on measurement of the impact of simulation teaching in emergency psychiatry [20].

The CAM-D consisted of 6 items, in which participants were asked to rate their confidence in key competency domains on a 4-point likert scale. These were:

1. Establish rapport with a patient who is acutely distressed
2. Take a history of mood disorder
3. Complete the mood aspect of a mental state examination
4. Complete a risk assessment of a patient with a mood disorder
5. Diagnose clinical depression
6. Form a basic management plan for someone with Depression.

The competency domains were derived from the undergraduate curriculum and were the learning objectives for the simulation exercise. They were deemed by faculty to be important components of a safe and complete assessment of a depressed patient. The CAM-D was used before and after the pilot session and was adapted slightly from a 5-point scale to a 4-point scale as students had a tendency to frequently rate themselves as the central ‘3’ on a 5-point scale. Each item on the CAM-D was scored, with a maximum score of 4 and a minimum score of 1. The maximum overall score for the CAM-D was 24, the minimum was 6. The internal consistency of the CAM-D was in the acceptable range (Cronbach’s alpha 0.702).

The CAM-D also included space for free-text in response to the questions; ‘Was there anything particularly good about the simulation teaching session?’ and ‘What would you change about the session?’. All the CAM-D questionnaires were anonymous, marked only with a number to enable matching of the pre and post questionnaires.

### Ethical approval

Ethical approval was gained prior to starting the study from the Joint Ethics and Research committee of Parirenyatwa hospital, Zimbabwe (JREC 04/13), the Medical Research Council of Zimbabwe (MRCZ/A/1728) and King’s College London Ethics committee (PNM/12/13-87).

Written consent was gained from all participants to use their anonymous confidence scores and written feedback in the study.

### Feasibility

23 out of a possible 30 (77%) students received the intervention, which was delivered by 3 postgraduate trainees and 3 nurses who had been trained to act as Simulated Patients.

Seven members of staff including 2 senior faculty and 5 postgraduate trainees were trained in facilitating the simulation sessions. Of these, 2 postgraduate trainees and one consultant delivered the intervention on this occasion.

5 members of the nursing staff received one-hour training sessions on acting as SPs, and of these, 3 took part as SPs during the sessions.

The two-hour session was introduced into the fourth-year undergraduate timetable and replaced time that students spent on clinical attachment to the wards. It was felt that this time spent away from clinical time was justified as they were exposed to presentations of depression and self-harm, which were not commonly seen on the inpatient psychiatric wards.

### Analysis

Using SPSS, version 20.0, confidence score data was plotted on Q-Q plots, which demonstrated a normal distribution. As such, a paired t-test was used to compare pre and post mean scores for each question on the confidence questionnaire.

Using SPSS, mean confidence scores were calculated for each domain and as an overall score. Pre and post scores were then compared using the paired t-test.

Free-text feedback was reviewed and summarised by two researchers (AP and MA). The major themes are presented below.

Out of a total number of 23 who took part, 2 students did not complete the questionnaires. One form had data missing and was included in the analysis, all others were complete.

Mean confidence scores increased significantly for each question on the questionnaire as shown in Table 1. The biggest increase in confidence was seen in ability to ‘Complete a risk assessment of a patient with Depression’ (mean difference +0.82, p = 0.001).

**Table 1:** **Confidence scores before and after simulation session and mean difference for each domain**

Rate your confidence in being able to: (n = 21)	Pre- session mean score (SD)	Post-session mean score (SD)	Mean difference	95% CI	t(20)	P value
Establish rapport with a patient who is acutely distressed	2.55 (0.67)	3.32 (0.65)	+0.77	0.41- 1.13	4.46	<0.001
Take a history of mood disorder	2.86 (0.60)	3.64 (0.49)	+0.77	0.44- 1.11	4.82	<0.001
Complete the mood aspect of a mental state examination	2.43 (0.56)	3.19 (0.61)	+0.76	0.41- 1.11	4.54	<0.001
Complete a risk assessment of a patient with a mood disorder	2.50 (0.96)	3.32 (0.48)	+0.82	0.39- 1.24	4.01	0.001
Diagnose clinical depression	3.09 (0.75)	3.68 (0.57)	+0.59	0.19- 0.99	3.05	0.006
Form a basic management plan for someone with depression	2.45 (1.01)	3.00(0.69)	+0.55	0.17- 0.93	2.98	0.007
Total score	15.90	20.05	+4.14	2.58- 5.71	5.52	<0.001

### Free-text responses

Out of a total of 21 questionnaires completed after the session, 18 completed the free text section.

Free-text responses were positive. Thirteen students commented that they found the feedback from peers and facilitators helpful, “criticism from others was very helpful”. Ten students commented that the experience was “relaxing” or “enjoyable” and six wrote that the simulation helped to “boost confidence”. Fourteen students felt that the simulation was relatively true to life; “it gave me an insight on what to expect with real patients and that all patients are not co-operative and you have to be flexible enough to go around it.”

In response to the question ‘What would you change about the session?’, four students commented that they would have liked smaller groups in order to ensure everyone had a chance to interview a simulated patient. Seven students also commented that they would have liked to have simulation teaching for other conditions as well as depression. These comments demonstrated that the students found the modality helpful and enjoyed the opportunity to practice interviewing skills in front of their peers and facilitators. There were no comments to suggest that students found this modality stressful or intimidating.

Seven students suggested using real patients as a way to improve the session and some commented that the simulated patients “were giving us answers to questions before we asked them” and that real patients “that will not volunteer information” would be better.

## Discussion

### Main findings

We have carried out adaptation of scenarios for simulated teaching in psychiatry for use in Zimbabwe. These were implemented by a team of one consultant and two post-graduate trainee psychiatrists with nursing staff trained in under one hour to act the scenarios. Over 20 students were trained on assessment and management of depression and suicide, by taking part in small groups to interview a simulated patient and then get structured feedback from peers and facilitators. Free-text feedback was positive overall with students commenting that it was “helpful”, “enjoyable” and “boosted confidence”. We showed that confidence increased significantly in all six domains measured compared to that measured before the teaching.

It seems that this intervention was feasible in a low-income setting and that, by involving postgraduate trainees in the delivery of the sessions, no additional demands were made on the small existing faculty in psychiatry. The use of nursing staff, who volunteered their time, meant that it was possible to deliver the sessions without incurring additional costs of actors. In addition, it offered students exposure to a wider breadth of psychiatric conditions than they would have experienced from clinical attachments alone.

We found that participation in the simulation session resulted in a significant increase in student confidence in assessing and managing depression. Our results compare to work done in high income countries which have demonstrated that undergraduates have rated simulation teaching in psychiatry as highly valuable and useful in improving clinical skills and increasing confidence [5,21]. This was the first study looking at simulation teaching in psychiatry in Africa.

All p-values of the mean differences in our study were less than 0.007, indicating that the change demonstrated is extremely unlikely to be due to chance. As all students undertaking their psychiatry placement at this time were included in the study, selection bias is unlikely although 7 students were unable to attend due to transport reasons. There is a possibility that students were more likely to report an increase in confidence and give positive feedback with facilitators remaining in the room whilst they completed their forms. However, all questionnaires were anonymous, which should have minimized this as a possible source of bias.

The positive free-text responses to our intervention indicated that simulation teaching increased student enjoyment as well as confidence. This replicates work done in high-resource settings where simulated patients have been demonstrated as being acceptable to students and preferable to role-playing scenarios with a colleague [22].

### Limitations

One limitation of our study is the lack of a control group. In future work, a control group could be included to compare confidence with those who had only undertaken the usual teaching program. This was not included in our study as the intervention was offered to all students on their psychiatry placement at that time.

Our intervention included a 20-minute didactic ‘refresher’ lecture. From our study, it is not possible to say that the increase in confidence scores was not due, at least in part, to the lecture rather than the simulation itself. However, this formed only a small part of the two-hour session, and revisited material already delivered in lectures earlier in the course.

Whilst our study demonstrated increased student confidence, we did not objectively measure the impact of the simulation on student competence. It has been demonstrated in some studies that self-assessment of confidence is directly correlated to student competence [23]. It is therefore likely that by improving student confidence, we have improved the skill base in recognising and treating depression amongst medical students in Zimbabwe. A way to measure this objectively would be to use structured OSCEs to compare performance of students who had taken part in the simulation with those who had not. This is planned as part of the future work of IMHERZ.

In addition, improved student confidence was measured directly after the intervention, however our study has not demonstrated that this effect is maintained over time. Further work would be required to assess whether the effect is sustained after students graduate and start practicing, one year later.

One area highlighted for improvement is the quality of the SPs, with a number of students commenting that the SPs appeared to 'give away' their symptoms without students having to ask them directly. The fidelity of simulated patients is particularly pertinent in psychiatry where subtle differences in affect and behavior can affect diagnosis and management. This is a significant problem when using nursing staff as SPs, although similar concerns have been expressed in literature from higher-income countries where actors are used [24]. This may be overcome by recruiting acting students, ex-patients who are currently stable or by further training of the current SPs on the importance of standardization.

Although our study demonstrated that this intervention was feasible on this occasion, this method of teaching is more resource-intensive due to the time of the nursing staff involved. This raises questions about the sustainability of this intervention in its current form. On this occasion, nursing staff were able to volunteer their time but this may not be possible long-term.

Following on from this intervention, a more sustainable method is for the students themselves to role-play the patients in the scenarios. This requires the student to spend an additional ten minutes during the session to read through the simulated patient brief and familiarize themselves with the case. This method obvious drawbacks in terms of moving away from the simulation model, but would be pragmatic and feasible. Another possible benefit of this method is increased empathy and reflection on the role of the patient by the student undertaking the role-play. These options are currently being explored as work on IMHERZ continues but is an area where limited resources may place low-income countries at a disadvantage.

### Why does it matter?

There are four principal reasons why this work is important; More than 13% of the global burden of disease is due to neuropsychiatric disorders, and almost three-quarters of this burden lies in low- and middle-income countries. Recent work demonstrated that 90% of people with mental illness in Africa receive no treatment [25]. A global mental health campaign was launched in 2007 to address this treatment gap and called for improved training of mental health professionals and access to mental health treatment in primary care [26]. Improved skills in recognition and management of mental disorders for doctors in Zimbabwe is essential in improving treatment but also for dissemination of knowledge to other health care personnel and to influence policy at a government level.

The problem of limited and variable student exposure to certain core psychiatric presentations is common across both high and low-income settings [2,27]. It is particularly important however to increase the skill base of all medical staff in low income countries such as Zimbabwe in order to address the large treatment gap in mental disorders [25]. Recent work in other African countries has demonstrated significant gaps in the skill base of general medical staff in recognizing and treating depression, and calls for improvement of undergraduate teaching on depression [28]. By involving nurses and psychiatry trainees, a teaching community across the mental health services in Zimbabwe was formed, which has resulted in better communication and sharing of ideas between different disciplines and grades.

Improving students’ enjoyment and engagement in psychiatry at undergraduate level has been shown to improve attitudes towards psychiatry [29]. This is important both in challenging stigma around mental illness [30] and in terms of recruiting doctors to the specialty [31]. Recruitment of junior doctors to psychiatry in Zimbabwe is critical in order to strengthen the mental health service and increase faculty numbers.

The experience gained by faculty and postgraduate trainees means that simulation can be developed to deliver experience in other clinical areas, for example, in training around a new diagnosis of HIV or management of high-risk patients in the forensic setting.

## Conclusions

Simulation is enjoyable and effective in improving student confidence in diagnosing and managing depression. Simulation techniques show promise as a feasible way of teaching medical students in a low-income setting and are a relatively simple way to increase the breadth of student exposure to different psychiatric conditions. Including postgraduate trainees as facilitators means that there is no increased burden on a small faculty. Training nursing staff to act as SPs offers an alternative where paying for actors is not possible. However, this poses difficulties in terms of sustainability, as they are required to take time away from their normal duties. Possible alternatives include the use of recovered patients, drama students or asking students to role-play the SPs.

Future work in this area should focus on the effect of novel teaching methods on clinical competence and on the effect on confidence in the longer-term.

## References

[1] Issenberg SB, Scalese RJ, Issenberg SB, Scalese RJ (2008). Simulation in health care education. Perspectives in Biology & Medicine.

[2] McNaughton N, Ravitz P, Wadell A, Hodges BD (2008). Psychiatric education and simulation: a review of the literature. Can J Psychiatry.

[3] Gorman PJ, Meier AH, Rawn C, Krummel TM (2000). The future of medical education is no longer blood and guts, it is bits and bytes. Am J Surg.

[4] McGaghie WC, Issenberg SB, Petrusa ER, Scalese RJ, McGaghie WC, Issenberg SB (2010). A critical review of simulation-based medical education research: 2003–2009. Medical Education.

[5] Hall MJMD, Adamo GMACMA, McCurry LMD, Lacy TMD, Waits WMD, Chow JMD (2004). Use of standardized patients to enhance a psychiatry clerkship. Academic Medicine Special Theme: Teaching Clinical Skills.

[6] Brown R, Doonan S, Shellenberger S (2005). Using children as simulated patients in communication training for residents and medical students: a pilot program. Acad Med.

[7] Tran T, Scherpbier A, Van Dalen J, Wright P (2012). Teacher-made models: the answer for medical skills training in developing countries?. BMC Medical Education.

[8] Jenkins R, Kydd R, Mullen P, Thomson K, Sculley J, Kuper S (2010). International migration of doctors, and its impact on availability of psychiatrists in low and middle income countries. PLoS One.

[9] Campbell C, Nhamo M, Scott K, Madanhire C, Nyamukapa C, Skovdal M (2013). The role of community conversations in facilitating local HIV competence: case study from rural Zimbabwe. BMC Public Health.

[10] Chibanda D, Mangezi W, Tshimanga M, Woelk G, Rusakaniko S, Stranix-Chibanda L (2010). Postnatal depression by HIV status among women in Zimbabwe. J Women’s Health.

[11] Abas M, Broadhead J (1997). Depression and anxiety among women in an urban setting in Zimbabwe. Psychol Med.

[12] Patel V, Araya R, Chatterjee S, Chisholm D, Cohen A, De Silva M (2007). Treatment and prevention of mental disorders in low-income and middle-income countries. Lancet.

[13] UNAIDS Zimbabwe [http://www.unaids.org/en/regionscountries/countries/Zimbabwe]

[14] Anonymous (2011). Health care: an African solution. Lancet.

[15] Abas MA, Nhiwatiwa SM, Mangezi W, Jack H, Piette A, Cowan FM (2014). Building mental health workforce capacity through training and retention of psychiatrists in Zimbabwe. International Review of Psychiatry.

[16] Mullan F, Frehywot S, Omaswa F, Buch E, Chen C, Greysen SR (2011). Medical schools in sub-Saharan Africa. Lancet.

[17] The MEPI network [http://www.mepinetwork.org/about-mepi.html]

[18] Extreme Psychiatry [http://www.extremepsychiatry.com]

[19] Bandura A (2006). Guide for constructing self-efficacy scales. Self-efficacy beliefs of adolescents..

[20] Thomson ABCS, Key S. Jaye P. Medical Teacher: Iversen AC How we developed an emergency psychiatry training course for new residents using principles of high-fidelity simulation; 2013 10.3109/0142159X.2013.803522 24006955

[21] Haeseler F, Fortin AH, Pfeiffer C, Walters C, Martino S, Haeseler F (2011). Assessment of a motivational interviewing curriculum for year 3 medical students using a standardized patient case. Patient Education & Counseling.

[22] Cleland JA, Abe K, Rethans J-J (2009). The use of simulated patients in medical education: AMEE Guide No 42 1. Medical Teacher.

[23] Regehr G, Hodges B, Tiberius R, Lofchy J (1996). Measuring self-assessment skills: An innovative relative ranking model. Acad Med.

[24] Krahn LE, Bostwick JM, Sutor B, Olsen MW (2002). The challenge of empathy: a pilot study of the use of standardized patients to teach introductory psychopathology to medical students. Academic Psychiatry.

[25] Kohn R (2004). The treatment gap in mental health care. Bull World Health Organ.

[26] Chisholm D, Lund C, Saxena S (2007). Cost of scaling up mental healthcare in low- and middle-income countries. Br J Psychiatry.

[27] Aguilera A, Garza MJ, Munoz RF (2010). Group cognitive-behavioral therapy for depression in Spanish: culture-sensitive manualized treatment in practice. J Clin Psychol.

[28] James BO, Jenkins R, Lawani AO (2012). Depression in primary care: the knowledge, attitudes and practice of general practitioners in Benin City. Nigeria South African Family Practice.

[29] Simmons M, Wilkinson P. Lectures versus case discussions: randomised trial of undergraduate psychiatry teaching. 2012

[30] Laugharne R, Appiah-Poku J, Laugharne J, Shankar R, Laugharne R, Appiah-Poku J (2009). Attitudes toward psychiatry among final-year medical students in Kumasi Ghana. Acad Psychiatry.

[31] Goldacre MJ, Fazel S, Smith F, Lambert T (2013). Choice and rejection of psychiatry as a career: surveys of UK medical graduates from 1974 to 2009. Br J Psychiatry.

